# Evolution of Regional Information Infrastructures Integrating Health and Social Care in Scotland: Qualitative Study

**DOI:** 10.2196/90997

**Published:** 2026-07-10

**Authors:** Varun Sai, Robin Williams, Kathrin Cresswell

**Affiliations:** 1 Usher Institute, University of Edinburgh, 5 Little France Road, Edinburgh, Scotland, EH16 4UX, United Kingdom, 44 0131 651 7869; 2 Institute for the Study of Science, Technology and Innovation, University of Edinburgh, Edinburgh, Scotland, United Kingdom

**Keywords:** health information exchange, information systems, intersectoral collaboration, delivery of health care, integrated, health information interoperability, United Kingdom

## Abstract

**Background:**

Expectations of integrating health and social care providers have driven the development of digital solutions aimed at overcoming interoperability challenges and ensuring access to information needed for integrated care across fragmented services. However, challenges persist in aligning diverse coding practices, heterogeneous data-sharing mechanisms, and stakeholder needs.

**Objective:**

We examine how expectations of interoperability and integrated care have shaped the growth of regional information infrastructures in Scotland, using the Key Information Summary (KIS), a summary record that shares key patient information from general practitioner records with out-of-hour services, ambulance services, hospitals, social workers, and caregivers across multiple care settings, as a case study.

**Methods:**

This qualitative study examined the development, implementation, and adoption of KIS in Lothian, Scotland, across health and care settings, where it has been in use for 13 years. Multisited ethnography was used to understand how technology design, implementation, and adoption were shaped by social, organizational, cultural, and political factors. Data were collected through interviews with users, vendors, and implementers; observations of technology use and multidisciplinary team meetings; and documentary analysis of policies, user guides, and internal reports. A hybrid analytical approach was applied: the Technology, People, Organization, and Macroenvironment framework guided initial coding, while the sociology of expectations and information infrastructure theory were used inductively to trace evolving visions of integration, and the long-term development of regional information infrastructure.

**Results:**

Data included 54 qualitative interviews, 20 hours of observation, and 59 documents collected between April 2024 and March 2025. Findings illustrate how information infrastructures for integrating health and care providers evolved through successive concerted efforts, conceptualized as waves. Three waves were identified, each characterized by attempts to interlink disparate information systems used by various health and care providers. The first wave focused on linking health care providers by developing networks and architectures required for sharing clinical information, which later supported the development and sharing of KIS. Subsequent waves sought to interlink information systems used by health care providers with those used by local authorities and social care providers. In the absence of shared data standards across these sectors, interoperability was achieved by extending the existing health care–centric infrastructure to different social care settings through workarounds such as providing proxy access to hospital systems and secure emailing networks.

**Conclusions:**

This work illustrates how regional information infrastructures for integrated care evolve through orchestrated waves of change. Some expectations for change required coordinated, system-level action, such as setting up standards, networks, and architecture, while others were realized through local adaptations. Integrating health and care providers through digitalization is a long-term process requiring sustained coordination, with progress often occurring through incremental, local extensions. Policies must support adaptive, long-term coordination, balancing system-level initiatives with local adaptations to achieve meaningful integration.

## Introduction

Digital technologies such as summary care records and shared care records (Textbox 1) are designed to facilitate the sharing of information between various professional and nonprofessional entities to integrate fragmented health and social care sectors [1-3]. In different health and care settings, these digital technologies for documentation and coordination coevolve with existing organizational structures, user skills, information-sharing practices, work routines, and legacy information systems, as they attempt to integrate the fragmented installed base of the information infrastructure [3,4]. Information infrastructures are sociotechnical bases that are embedded into everyday routines and users’ needs in the form of information systems and practices [3,5,6]. Information infrastructures for integrating health and social care sectors emerge from evolving efforts to interlink the different information systems used by diverse professional entities (Ibid). As a result, information infrastructures are not created from scratch*,* but rather grow over extended periods across multiple settings, building upon the preexisting environment of information systems, users, and their information needs [4,5].

Textbox 1. **Definitions of technologies, types of care provisioning, and interoperability discussed in this study.**

Definitions

1. Electronic health record (EHR): EHRs provide a comprehensive, longitudinal view of a patient’s health journey across multiple health care settings [7].
2. Summary Care Record: the Summary Care Record is a national database that holds important patient information such as current medication, allergies, and details of any previous bad reactions to medicines, created from general practitioner (GP) medical records [8].
3. Shared Care Record (ShCR): ShCR is a safe and secure way of bringing information from an individual’s separate records from different health and care organizations together digitally in one place [9]. While the ShCR program is nationally defined, ShCRs are delivered as regionally commissioned and governed systems rather than a single UK-wide record.
4. SNOMED CT is a comprehensive clinical health care terminology that enables standardization of clinical terminologies in EHRs [10].
5. Health Level Seven (HL7): a set of international standards for the exchange of data among health care computer applications [11].
6. OpenEHR: OpenEHR provides technical standards for an EHR platform, along with domain‑developed clinical models to define content [12].
7. Integrated care: this is a worldwide trend in health care reforms and new organizational arrangements focusing on more coordinated and integrated forms of care provision between health and care providers [13].
8. Patient-centric care: care is personalized, coordinated, and enabling so that people can make choices, manage their own health, and live independent lives, where possible [14].
9. Technical interoperability: this is the ability of systems and services to connect and communicate through common technical standards, interfaces, data exchange methods, and secure communication protocols [15].
10. Semantic interoperability: this is the ability of different systems to exchange data with a shared and accurate understanding of its meaning through common vocabularies, schemas, and information management strategies [15]
11. Organizational interoperability: this is the ability of different administrative entities to align business processes, cooperate effectively, and establish clear relationships to deliver user-focused and accessible public services [15].
12. Legal interoperability: this is the ability of public administrations operating under different legal frameworks to work together in delivering services by identifying and addressing interoperability barriers, ensuring coherence between legislation, assessing the impact of information and communications technology, and maintaining the legal validity of data exchange and data protection across borders through additional agreements where necessary [15].

Information infrastructures grow as expectations and imagined ways (imaginaries) of integrating disparate information systems used by health and care providers evolve, driven by efforts to interlink interfaces of different information systems and to standardize data sharing [16-18]. Imaginaries, for this study, are conceptualized as the collective visions and ideas of different stakeholder groups in interlinking information systems used by different health and social care providers. But these efforts to interlink disparate information systems are disrupted by the emergence of new technologies, workflows, and increasing demands for integrated care. Despite persistent challenges in achieving practical information exchange between disparate systems, a generic vision of interoperability has endured, extending beyond and being continually reshaped by successive attempts to build shared information infrastructures. These imaginaries have coevolved with emerging expectations of providing integrated care, where interoperability is perceived as a vehicle for enhancing and delivering integrated care by developing common standards and semantics for interoperable communication [16-18]. In the domain of the sociology of expectations in science technology studies (STSs), scholars have identified how expectations are performative and attract the interests of allies, leading to market coordination, research and innovation, and shaping social action [19-21]. Expectations are also temporal and iterative, shifting over hype cycles between optimism and disillusion; they vary between social groups due to differences in values and trust; and they have material effects in the form of artifacts and actions promising to address persistent challenges [22].

Scholars examining technological development through the lens of hype cycles emphasize that technological development and innovation unfold progressively, moving from initial conception through phases of inflated expectations to eventual stabilization and adoption [19-22]. Existing research has examined the development of health care information infrastructures through modular approaches, whereby new functionalities are layered onto existing systems and interfaces are extended to accommodate additional actors [3-6]. In parallel, scholars have emphasized the role of expectations in shaping innovation by mobilizing actors and resources around shared visions [19-22]. However, there is limited understanding of how information infrastructures that interlink both health and social care evolve, and how expectations shape this process. This study addresses this gap by examining how these theoretical perspectives apply within efforts to support information sharing and coordination across health and social care organizations. This gap is significant given the complexity of enabling interoperability and coordination across diverse organizations with differing cultures and professional identities, where expectations shape stakeholder engagement, power dynamics, and implementation outcomes [23]. Expectations in this regard are not only discursive but also materialized through the design and development of information infrastructures intended to support interoperability across systems [24].

Achieving interoperability and interoperation between diverse clinical and nonclinical entities requires the development of shared standards across health and care institutions (Textbox 1), which can bridge heterogeneous technological systems and the differing coding practices embedded within their various local legacy systems [2,17]. The initial quest for interoperability materialized in a series of technical initiatives, including the development of grids and gateways to enable secure electronic communication between various health care providers, followed by efforts to standardize clinical terminologies through initiatives such as SNOMED CT to ensure consistent coding and interpretation of health data, reflecting an early focus on achieving semantic interoperability [25,26]. As expectations around linking different service providers evolved to provide integrated care and patient-centric care, standards such as Health Level Seven (HL7), focusing on exchanging health data between information systems, and OpenEHR to structure and model health data to ensure semantic consistency across various health and care settings were developed [26]. However, interoperability in this study is understood not merely as a technical and semantic property [27,28], but as a sociotechnical process causing an interplay between technical systems and the social and organizational practices in which they are embedded, including workflows, professional roles, and coordination between stakeholders [17]. Interoperability requires aligning networks and architectures for information sharing across disparate systems, alongside differing coding practices, care routines, information governance arrangements, and stakeholder responsibilities, reflecting the interconnected technical, semantic, organizational, and legal layers of interoperability [15,17]. Standardizing practices and achieving interoperability between different health and care entities involves various sociotechnical and socio-organizational challenges. For example, users and organizations may fail to use or abandon new technologies that are incompatible with their specific local data collection practices and routines, or they may develop alternative modes of use that diverge from intended use [29]. In this study, attention is primarily given to technical and semantic interoperability, while considering their embeddedness within broader sociotechnical, organizational, and legal contexts [15,17].

Countries around the world have spent decades attempting to integrate health and social care services through digitalization, involving significant institutional, legal, and technological changes, mobilizing the interest of various stakeholder groups [30-32]. In this regard, Scotland has established a relatively streamlined approach to the integration of health and social care services by forming stable regional Integrated Joint Boards (IJBs) as a part of The Public Bodies (Joint Working) (Scotland) Act 2014 and by providing national guidelines to suppliers for developing digital technologies [33,34].

The Key Information Summary (KIS) in Scotland is an electronic health record (EHR) that has been used for over a decade to extract and share key patient information from general practitioners (GPs) to other health and care providers. As an information infrastructure, the KIS is an extension of the emergency care summary (ECS) introduced in 2006, which is a national-level information infrastructure for sharing data held in information systems used by GPs with other primary and secondary care providers (Textbox 2).

Textbox 2. **Key information summary in Scotland.**

Key Information Summary (KIS) in Scotland

Implemented in 2012, the KIS in Scotland is an extension to the emergency care summary (ECS), and shares information held in the general practitioner (GP) records with out-of-hour services, the Scottish Ambulance Service, hospital specialists, social workers, and caregivers. The ECS contains demographic details, active and acute medication issued by the GP, allergy data, and the KIS if recorded. The KIS builds on the ECS database and includes information such as the past medication history, baseline functional and clinical status, current care and escalation plans, emergency contacts and next of kin details, patient's preferred place of care, and resuscitation status. The KIS also includes a “special notes” section to record additional information relevant to future care planning. It allows clinicians to add free-text information that highlights contextually important care needs that may not be captured through structured coding, supporting more comprehensive Future Care Planning. KIS is automatically shared from the GP's computer system twice a day, and is stored at a National Health Service (NHS) data center, where a read-only version is made available to other users. GPs access KIS through the Vision and Egton medical information systems, which grant them administrative rights. Out-of-hour services use the Adastra information system, hospitals use the TrakCare information system, and the Scottish Ambulance Service uses the Terrafix information system, each providing read-only access to the KIS. Social workers from local authorities and social care providers are provided with a physical copy of the KIS, which is shared directly by GP practices.

Health and Care Provisioning in Scotland

In Scotland, health and social care services are coordinated regionally by 14 NHS health boards and 32 local authorities by forming Integrated Joint Boards (IJBs), following the Public Bodies (Joint Working) (Scotland) Act 2014. IJBs coordinate the planning and delivery of integrated health and social care services. GPs are responsible for delivering health care services at the primary care level during daytime hours, while out-of-hour services manage urgent health care needs outside of these times. Hospitals provide care at secondary and tertiary levels under the oversight of the health board. Local authorities, also known as councils, are responsible for delivering social work services, including adult social care assessment, safeguarding, care coordination, and support for vulnerable populations. In the Scottish context, social care refers to nonmedical and community-based support services that assist individuals with aging, disability, long-term conditions, mental health needs, and independent living. These services include home care, residential and nursing care homes, rehabilitation support, and community-based support services. Social care provision is delivered through a mixed landscape comprising NHS health boards, local authorities, private providers, and voluntary or third-sector organizations. Following the 2018 General Medical Services Contract, GPs, hospital-based health specialists, social workers from local authorities, and care workers collaborate within multidisciplinary teams and care pathways to deliver integrated care. The health boards and local authorities receive financial allocations from the Scottish Government, which are subsequently pooled and managed to operate the IJBs.

**Figure 1.**
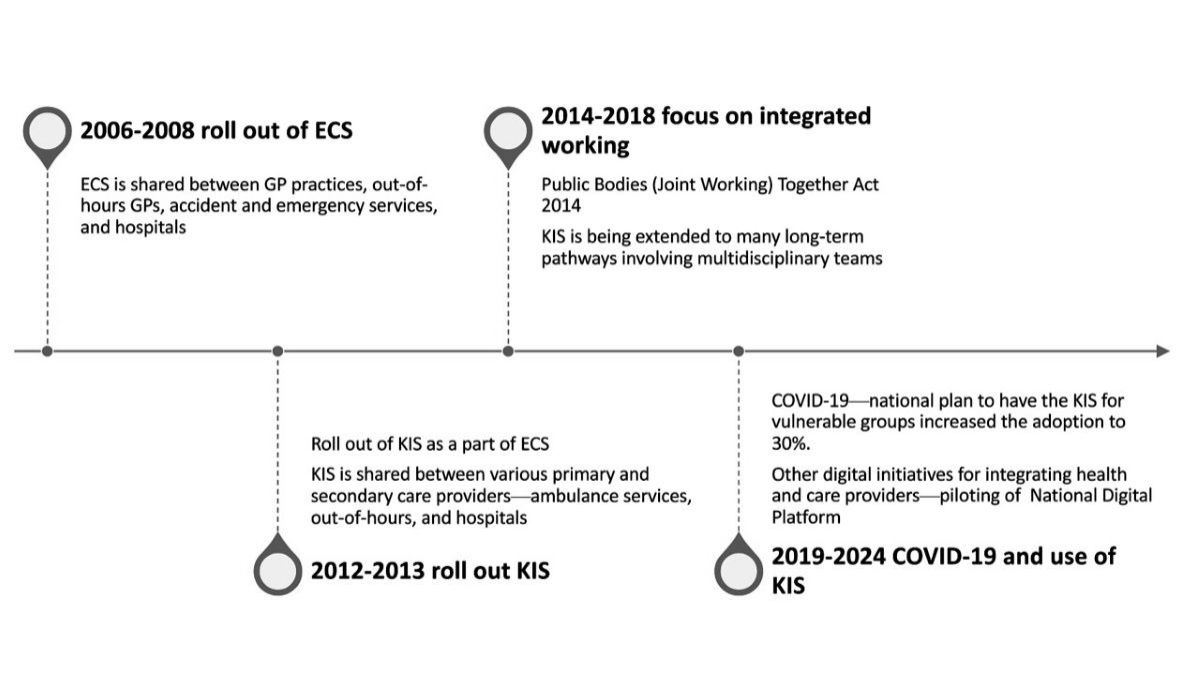
Timelines regarding rollout of ECS and KIS. ECS: emergency care summary; GP: general practitioner; KIS: Key Information Summary.

Figure 1 illustrates the timelines of the rollout of ECS and KIS in Scotland. From its introduction in 2012, the KIS has been implemented and adopted across multiple long-term and end-of-life pathways to share up-to-date key information, as expectations around interoperability and integrated care evolved. Unlike hospital- or GP-based EHRs, which are designed for use within specific provider organizations, the KIS functions as a summary record intended to support information sharing across multiple organizations and professional groups, depending on the care pathways in which an individual is involved. During COVID-19, the KIS was repurposed to identify vulnerable patient groups, which increased its adoption [35]. While several parallel digital initiatives (such as a national-level platform) have sought to enable information sharing across various health and care providers in Scotland, the KIS remains the only EHR shared between health and social care sectors. Although in use for over 13 years as a national-level initiative, the KIS varies significantly in its use across regions, and this variation remains largely unexplained. Similarly, the processes through which some regions have extended and adapted the use of the KIS over time, including how new actors and professional groups become enrolled within the evolving information infrastructure, remain underexplored. Expectations play a key role in this process, as initial visions and promises are continually revised and revisited over time, generating renewed ambitions for health and social care integration through efforts to enable interoperability. However, the role of collective imaginaries across different entities in the integrated care system, the variations and similarities among them, the mechanisms through which new actors are incorporated, and their effects on the evolution of the information infrastructure remain unexplored. The KIS, building on the ECS as an information infrastructure, offers an opportunity to trace and understand how such infrastructures evolve, as they are assigned new purposes and extended to new actors in response to evolving expectations around integrated care and interoperability. In doing so, it offers insights into how long-term integration efforts enabling interoperability unfold in practice, and how such processes may be more effectively coordinated across heterogeneous organizational and regional contexts.

This paper aims to explore how expectations and imaginaries have shaped the evolution of regional information infrastructures, using Scotland’s KIS as a case study to examine the trajectory of health and social care integration through digitalization across different time periods.

## Methods

### Setting: The KIS in Scotland

**Figure 2.**
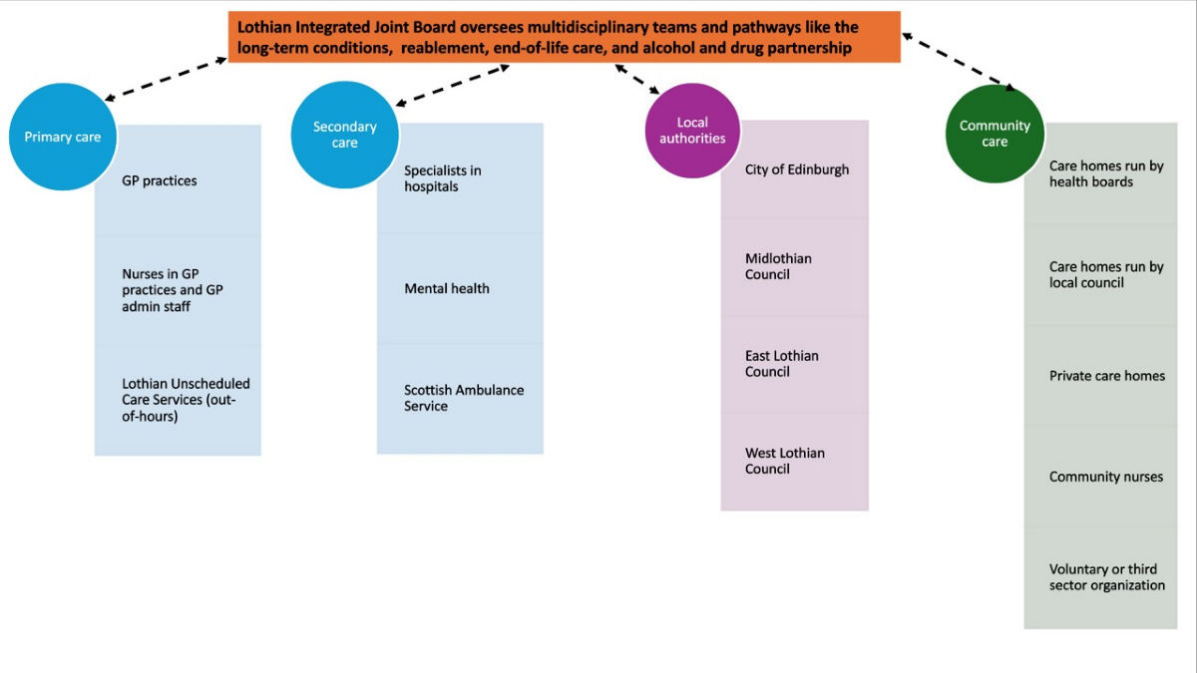
Health and care provisioning in Lothian. GP: general practitioner.

The implementation, adoption, and use of the KIS vary across regions, and the IJB of Lothian, a region in Scotland, serves as an exemplar in using the KIS for Future Care Planning by linking different health and care entities using diverse information systems. In Lothian, NHS (National Health Service) Lothian and the four local authorities (East Lothian, West Lothian, Midlothian, and Edinburgh City), also referred to as councils, form the IJB, directing the course of integrated care regionally, as illustrated in Figure 2.

The KIS is not only used by clinicians linked to NHS Lothian, such as GPs, out-of-hours staff, nurses, specialists, and paramedics, but also by social workers from local authorities, community workers, and social care workers in a few care homes. At the national level, the KIS is neither consistently shared with social workers and care providers, nor do they have the means to share information with the GPs using the KIS. However, unlike other health boards, NHS Lothian developed separate templates for community nurses and care workers to share information with GPs, resulting in the KIS taking multiple material forms, including PDFs and paper records, as expectations of providing integrated care evolved. The implementation of the KIS in Lothian was chosen to examine how expectations and imaginaries have shaped the growth of regional information infrastructures within preexisting local contexts, including established information-sharing practices, routines, and the use of diverse information systems across fragmented health and social care organizations in Scotland.

### Study Design

This multisited longitudinal ethnographic study used the Biography of Artifacts and Practices (BoAP) [36] and Technology, People, Organization, and Macroenvironment (TPOM) frameworks [37]. Multisited ethnography is a methodological approach that encourages researchers to observe and study the multiple sites and settings in which a technology is designed, implemented, adopted, and evolves over an extended timeframe [38]. BoAP was developed to provide researchers with a methodological tool to navigate various spaces of technological development and adoption, enabling them to reflect on and articulate their choices of settings, periods, and focus of inquiry.

TPOM is a theory-informed evaluation framework designed to capture the various sociotechnical, socio-organizational, and macroenvironmental factors affecting technology implementation, adoption, and use. While the TPOM framework functions as an evaluation framework for examining digital technologies in real time, the BoAP framework is primarily used in longitudinal analyses to investigate how technologies are designed, developed, implemented, and used across diverse settings and temporal contexts. Accordingly, the two frameworks are complementary and help capture immediate and historical factors shaping technological development, implementation, adoption, and evolution. The development, implementation, and adoption of the KIS were examined over time across various health and care settings, with BoAP helping reflect on the settings and moments of study, and TPOM guiding data collection to explore factors shaping technology development, implementation, and use. Qualitative methods, including interviews, ethnographic observations, and documentary analysis, were used in this study to explore the sociotechnical, socio-organizational, and macroenvironmental factors shaping the development, implementation, adoption, and use of the KIS in Lothian, within the broader trajectory of integration in Scotland.

### Ethical Considerations

Research approvals from the Edinburgh Medical School Research Ethics Committee were obtained on April 17, 2024, and June 17, 2024, for conducting interviews (24-EMREC-012) with participants in Scotland and the United Kingdom, and for observing (24-EMREC-043) meetings, events, and training sessions related to technology use. This study was also approved by the quality improvement team of NHS Lothian. Informed consent was obtained from all participants before their participation in this study. To protect participant privacy and confidentiality, all identifying information was removed from the data, and participants were anonymized in all study records, analyses, and reporting. Participation was entirely voluntary, and participants were free to decline or withdraw from this study at any time without consequence. No compensation, incentives, or payments were offered for participation.

### Sampling

BoAP and TPOM guided the sampling of participants for this study, encompassing professionals from health and social care settings, social workers, policymakers, technology designers, academics, and implementation scientists. These approaches were selected as information infrastructures grow over extended periods, making it necessary to examine multiple historical time frames and perspectives of diverse actors who conceived, developed, implemented, and used the systems [36,38]. BoAP’s longitudinal, reflective lens supported tracing KIS’ evolution and identifying relevant settings and actors central to its development, implementation, and use. TPOM, with its focus on elucidating technological, sociotechnical, socio-organizational, and macroenvironmental factors, was useful in identifying participants across micro, meso, and macro levels engaged in the implementation, adoption, and use of the KIS.

Purposive sampling and snowballing were used to recruit participants. Purposive sampling enabled access to senior-level participants who, through their involvement in the conceptualization, implementation, or adoption of the KIS at different time periods, were uniquely positioned to provide specialized, context-rich insights. The gatekeepers, namely, the quality improvement team of NHS Lothian and the out-of-hour service run by Lothian Unscheduled Care Services, were approached to help identify and recruit potential participants. Regarding observation, gatekeepers were approached to identify and observe stakeholder meetings, multidisciplinary team (MDT) meetings where the KIS was used for Future Care Planning, and training sessions on using the KIS in various health and care settings. MDTs relying on the KIS to coordinate Future Care Planning included GPs, hospital specialists, community teams, out-of-hour services, local authority social workers, and managers from a small number of care homes. For interviews, participants representing varying health and social care settings, including all four councils of Lothian, were sampled. Policymakers in the domain of health and social care, identified through national policy documents, and academics studying information systems, were purposively sampled to explore the macroenvironmental factors and the changing policy landscape. International and national technology vendors were included purposively to understand factors underpinning technological design, development, and support. Snowballing was used by asking interview participants if they would consider suggesting potential participants who were involved in the development, implementation, adoption, and use of the KIS.

### Data Collection

Data was collected over 11 months from April 2024 to March 2025 using interviews, ethnographic nonparticipant observations, and documentary analysis. Data collection continued until saturation, that is, until no new perspectives regarding the development, implementation, adoption, and use of the KIS emerged.

One-to-one, semistructured interviews were conducted with implementers, policy leads, data analysts, academics, technology developers, and health and social care providers in Lothian, Scotland, to capture their perspectives around developing, implementing, adopting, and using the KIS. Interviews, lasting 40‐60 minutes, were conducted and recorded online using Microsoft Teams, transcribed, and checked for accuracy.

Interviews, guided by the TPOM framework [36], aimed to elicit discussions around the technological, sociotechnical, socio-organizational, and macroenvironmental factors affecting the implementation, adoption, and use of the KIS. Separate topic guides for implementers, users, vendors, academics, and policy leads were developed to gather insights into their specific roles and perspectives (Textbox 3).

Textbox 3. **Topic guide for interviews.**

Topic guide for the following:

1. **Academics, data analysts, and policy leads**
  The questions focused on the information systems in place before Key Information Summary (KIS) to better understand the rollout, introduction of KIS, initial expectations, comparable initiatives that informed its rollout, use, and interoperability with other technologies, the composition and digital maturity of initial users, and the extent of access to shared information across different settings, hype, and futuristic expectations around integrating health and social care using digital technologies.
2. **Implementers of KIS**
  The questions aimed to explore the implementer's role and the initial stakeholders involved in implementing KIS, their expectations and incentives, perceived changes in the technological and policy landscape over time, evolving expectations and responses to them, mechanisms supporting wider implementation, views on the long-term sustainability of the technology, and reflections on both successes and areas for improvement from implementing KIS.
3. **Vendors of digital technologies to integrate health and social care in Scotland**
  The questions explored vendors' involvement with KIS, initial expectations and experiences during its development and implementation, perceived facilitators and barriers, insights from comparable initiatives, interoperability with other technologies, shifts in the supplier landscape, mechanisms supporting ongoing implementation across new services, views on long-term sustainability of KIS, and reflections on both successes and areas for improvement in KIS.
4. **Users in different settings to understand the adoption of KIS**
  The questions examined how adaptable KIS was to the participants’ roles and settings, composition and digital maturity of users, the challenges and impacts on daily work practices, experiences of cross-provider communication, mechanisms for shared learning and support, organizational knowledge retention, and the influence of evolving policies and technological changes on the adoption process.

Nonparticipant observations were used to understand the working of MDTs and national planning groups in using the KIS for Future Care Planning. MDT meetings between NHS Lothian and the Edinburgh Health and Social Care Partnership (EHSCP), and national planning group meetings were observed to gain a deeper understanding of the nature of interprofessional working between different health and care entities in using the KIS for Future Care Planning at a regional and national level. All observations were conducted online via Microsoft Teams. Field notes were systematically recorded using an observation protocol (Textbox 4), with each session lasting between one and two hours.

Textbox 4. **Protocol for observation.**

**Purpose of the event**

- What is the purpose of the event or meeting, and what are the resources shared to provide context or information?
- Who is the organizer, and what references are being made to Key Information Summary (KIS)?

**Description of the actors and what they do**

- Job title or affiliation of the actors involved.
- Nature of their role in context of the meeting

**Insights into the process—use and adoption of the KIS**

- What are the references being made in terms of the use and adoption of the KIS and by whom?
- What resources are being used to share learning across participating organizations, and how is it shared?
- What future plans are being made in reference to the use and adoption of KIS?

**Insights into outcomes or impacts—practice or skills, workflow, and behavior or attitudes**

- How are participating institutions or organizations referring to the implementation and use of the KIS in the meeting or event?
- What problems are being reported and addressed?
- What problems are not being reported and addressed?
- What sources of information or documents are being shared?
- Is there a good rapport among participants? If so, among whom?

**Reactions of actors to specific questions**

**Feelings—researcher’s own impressions or feelings in relation to the observation**

Use of KIS by clinicians was observed to view how the KIS was used in practice. Observations also helped understand the dynamics at play between regional and national stakeholders in implementing and scaling the use of KIS, agreements, and tensions that emerged between different stakeholder groups.

Publicly available documents were retrieved from NHS and government websites using Google searches (eg, “Key Information Summary in Lothian and Scotland”) to identify relevant stakeholders. Participants were asked if they considered sharing any relevant documents regarding the development, implementation, adoption, and use of the KIS. Documents provided by users, vendors, implementers, and policy leads included internal case studies, evaluation and measurement plans, user guides, notifications of software updates, contingency plans, and archived websites containing information regarding the rollout of the KIS for GPs. National policies from the Department of Health and Social Care, and regional-level strategies from health boards and IJBs were collected to explore the similarities and contentions between national goals and regional narratives. These documents were collected to understand the historical and persistent challenges surrounding the KIS in various settings at different phases, and the broader trajectory of health and care integration in Scotland.

### Data Analysis

NVivo (version 14; Lumivero) was used to collate and code interviews, observation notes, and documents. Analysis followed an interpretative approach that acknowledged the researcher’s subjectivity. Validity and credibility were enhanced by seeking disconfirming evidence and data triangulation. Data triangulation involved converging all the data collected using different qualitative methods to contextualize the case study within the broader trajectory of health and care integration. That is, policy statements, observation notes, and interview data were analyzed to examine how expectations surrounding integrated care and interoperability shaped the material and performative enactment of the KIS, as well as the sociotechnical, organizational, and macroenvironmental factors influencing its development, implementation, and use.

Data analysis included an abductive approach, combining both inductive and deductive methods. Deductive thematic coding, guided by the TPOM framework, was applied to documents, interviews, and observational notes to understand the technological, sociotechnical, socio-organizational, and macroenvironmental factors influencing the development, implementation, adoption, and use of the KIS [36]. Theories and concepts from STS on information infrastructures [3-6] and sociology of expectations [19-22,25,39] informed inductive analysis to explore how expectations and imaginaries have shaped the growth of regional information infrastructures to integrate health and social care in Scotland, as illustrated in Table 1.

**Table 1.** Codes informing thematic analysis.

Aggregate themes	Second-order themes and abductive codes
First wave of interoperability	Epistemic codes, standards, and siloed digitalization Historical factors shaping the conception and development of the KIS^a^ Information sharing practices during the roll-out of ECS^b^ GPASS^c^ rollout in Scotland Incentives for GPASS adoption Coding practices in hospital settings Early visions of interoperability and health care–centric integration efforts Historical factors shaping the conception and development of the KIS Expectations of integrating health care providers Building a national information infrastructure Installed base-related challenges in developing KIS Technology procurement in different health care settings Historic issues around information sharing and seeking consent to share information Developing consent models for ECS and KIS Macroenvironmental and socio-organizational factors influencing the development and implementation of the KIS National guidelines to promote interprofessional working and integrated care National aspirations to integrate health and care providers digitally, and resulting performative actions to standardize information coding and sharing Shrinking vendor space, reduced scope for customization, and the continuing quest for interoperability Macroenvironmental and socio-organizational factors influencing the development and implementation of the KIS Interoperability guidelines to reduce vendor heterogeneity National guidelines for linking health care providers and local councils using digital technologies Networks of primary care providers assisting the digital transformation of primary care providers
Second wave of interoperability	Aspirations to link health and social care settings Technological, sociotechnical, and socio-organizational factors affecting the implementation and adoption of KIS Digital and nondigital material forms of KIS to enable information sharing among health and care providers Expectations of users (GPs^d^) in using the KIS for future care planning Expectations of other clinical users in using the KIS for future care planning Information desired for future care planning Desirability of extending the digital version of the KIS to care settings Digital transformation of local authorities Partial digital transformation of social care providers Trustworthiness of users and workarounds to provide access to information systems Technological, sociotechnical, and socio-organizational factors affecting the implementation and adoption of KIS Workarounds to extend the KIS to clinical settings Workarounds to extend the KIS to nonclinical settings Risk and trust in information sharing practices Communities sharing good practices for information sharing using the KIS Technological, sociotechnical, and socio-organizational factors in using the KIS for Future Care Planning Diverse care providers and workforce issues Incentives for GPs and GP practices to update the KIS Training care providers in using the KIS by health and social care partnerships Practice-based differences in updating the KIS Varied understandings of Future Care Planning in social care settings Alternative sources of information capturing the Future Care Plan in Social Care Settings Cultivating a sense of ownership in nonclinical settings
Third wave of interoperability	Local differences, national aspirations, and the expectations of having updated information Macroenvironmental, socio-organizational, and sociotechnical factors influencing adoption, scale, and future of the KIS Top-down approaches during the COVID-19 pandemic to identify vulnerable groups Increased adoption of the KIS during the COVID-19 pandemic Regional attempts in linking health care providers and local councils using the KIS before the COVID-19 pandemic Reduced information quality with the KIS stating “created as a part of the COVID-19 protocol” Increased workload to identify useful KIS Identifying key information in the KIS Aspirations of having an integrated platform and freeing data from legacy systems Macroenvironmental, socio-organizational, and sociotechnical factors influencing adoption, scale, and future of the KIS Parallel imaginaries of having a person-centric platform at the national level Parallel attempts to integrate health and care providers using digital technologies Asynchronous vendor contracts across regions in Scotland Asynchronous vendor contracts across service providers

a KIS: Key Information Summary

b ECS: Emergency Care Summary

c GPASS: General Practice Administration System for Scotland

d GP: General Practitioner

Recurring patterns and narratives around interoperability and integrated care were temporally grouped to capture how these expectations and imaginaries reappeared across successive “waves,” which formed the core theme guiding this study’s analysis. Within each wave, subthemes were identified by comparing the different imaginaries, epistemic coding practices across settings, local and national priorities, and examining how user needs were configured in standardized systems. Theories and concepts from STS on information infrastructure were used to map the installed base and tensions between the standardization and customization of digital technologies, while theories from the sociology of expectations informed the analysis of the material and performative effects of evolving expectations. This approach allowed for an examination of how long-term, utopian visions are gradually realized by mobilizing resources, aligning stakeholders, and breaking broad ambitions into short-term, actionable objectives.

## Results

### Overview

Data to inform the case study included 50 qualitative interviews (Table 2), notes from 20 hours of observation, and 59 documents (Table 2).

The various imaginaries and expectations highlighted through policies and guidelines to integrate different health and care entities were categorized as different waves of interoperability. These waves exhibited a temporal resurgence, with each wave problematizing the constraints imposed by existing data-sharing practices and seeking to interlink health and care providers using interoperability as a key mechanism. Three waves were identified; the following sections will describe the concept of waves, the different waves of interoperability, and the subthemes within them in depth.

**Table 2.** Dataset.

Methods (datasets) and descriptions/characteristics	Count, n
Interviews (50 interviews, each lasting 40‐60 min)	
GPs^a^ from daytime practices	14
Social workers and social care workers	13
Out-of-hour service	5
Data analysts from Public Health Scotland	4
Clinical informatics and population health experts	4
Technology vendors and software developers	4
GP admin staff	2
Government representatives for digital health and social care	2
Academics	2
Observations (12 meetings lasting 1‐2 h each and 20 h in total)	
Observing national-level collaborative meetings to use digital technologies for Future Care Planning from May 2024 to December 2024	8 h
Observing events hosted by social care organizations	8 h
Observing technology use by clinicians	4 h
Documents (59 documents and 2 websites)	
Meeting notes from care home meeting with GP practices for service improvement of future care planning	12
The KIS^b^ user guide for GPs, nurses, social workers, and social care workers	11
Information leaflets issued by various departments promoting the KIS	8
User guides and contingency plan for Vision and EMIS^c^ used by GPs, TrakCare used in hospital settings, and Adastra used in out-of-hour settings	8
National and regional information sharing guidelines, regulations, and policies	5
Economic impact measurement plans and assessments	4
Documents pertaining to seven steps to ACP^d^ for care home staff	4
Digital health and care strategies in Scotland	3
Internal case studies were around the introduction of the KIS in care homes in the Northeast of Edinburgh	2
Internal case studies about the use of the KIS by Alzheimer Scotland Post Diagnostic Dementia Service in Edinburgh, and by Reablement and End of Life Care in the Northwest of Edinburgh	2

a GP: general practitioner.

b KIS: Key Information Summary.

c EMIS: Egton medical information system.

d ACP: anticipatory care planning.

### Waves of Interoperability

**Figure 3.**
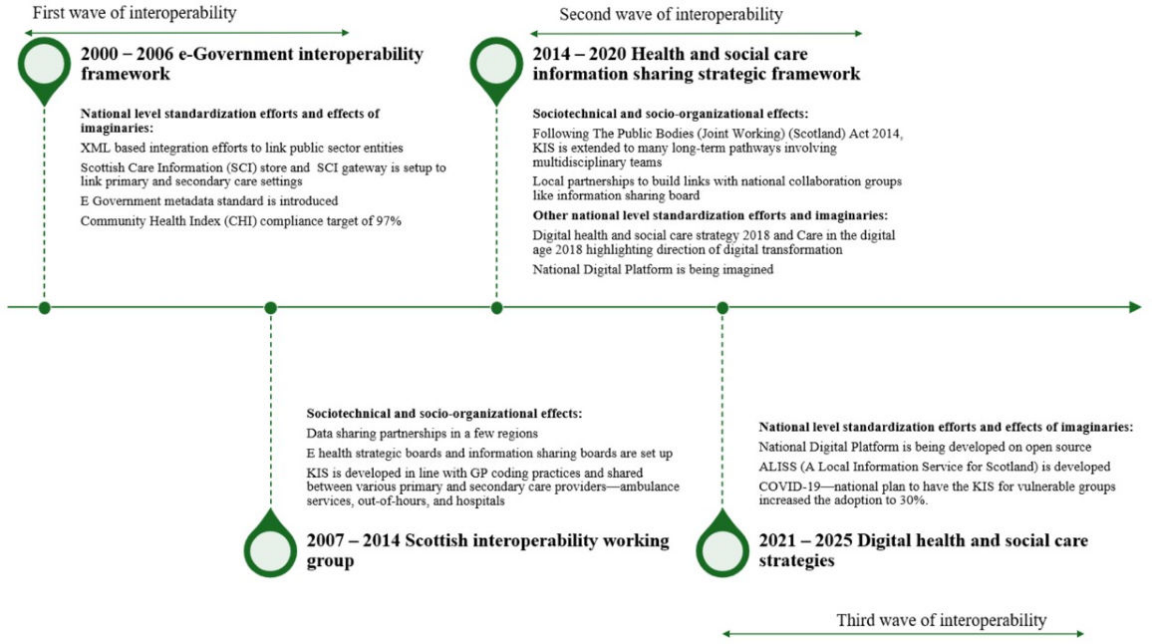
Evolving visions of interoperability. ALISS: A Local Information Service for Scotland; SCI: Scottish care information; CHI: community health index. KIS: Key Information Summary.

Figure 3 illustrates how interoperability and expectations of having interoperable systems in the integration of health and care services were conceptualized as three waves based on the imaginaries of integration.

These waves emerged not from a formalized planning process with fixed timelines but as new imaginaries of interoperability shaped the evolving data strategy and policy landscape. This, in turn, drove the development and implementation of new digital technologies, transforming routines and practices, and laying the foundation for further efforts to integrate health and care services through digitalization. Imaginaries, as collective visions of stakeholders, drove the initiation of policy directives and mobilized relevant actors and resources. These imaginaries were iterative, resurfacing with renewed expectations to connect and align existing information systems across health and care providers. Such expectations mobilized responses that crystallized into technological systems and practices, representing what was seen as achievable at the time in advancing interoperability, and in turn extended and reconfigured the installed base.

The first wave shown in Figure 3 emerged from efforts to digitalize health care services and interlink primary and secondary care services. This wave reflected an emerging national imaginary of interoperable health care systems, where patient information could be seamlessly shared across health care settings. Aligning to these expectations, the e-Government Interoperability Framework 2006 and Scottish Interoperability Working Groups 2007 were developed [39,40], aiming to streamline coding and data sharing practices across various health care entities, to achieve technical and semantic interoperability. The first wave included the conceptualization and piloting of the KIS as an early materialization of these interoperability ambitions, shaped by what was considered technically and organizationally feasible at the time. At the semantic level, as coding practices varied across health care settings, interoperability efforts using the KIS centered on sharing information in line with how GPs coded health data, using GP coding practice as a common reference point to minimize variability between systems, illustrating how existing practices within the installed base shaped the form that interoperability could take.

The second wave shown in Figure 3 aimed to connect health care systems with council systems and to digitalize social care providers, extending initial expectations of interoperability ambitions beyond health care settings. This reflected a renewed national imaginary of integrated health and social care systems linking health care providers, local authorities, and social care providers in the delivery of integrated care. The second wave, initiated by The Public Bodies (Joint Working) (Scotland) Act 2014 and the Health and Social Care Information Sharing Strategic Framework 2014, included attempts to link information systems used by social workers in local authorities and health care providers, alongside efforts to digitalize social care providers through various Digital Health and Social Care strategies [41,42]. The second wave included the implementation and use of the KIS for Future Care Planning in various health and care settings across different regions, representing a further iteration of interoperability efforts building on earlier developments as expectations of integrated care evolved, highlighting the challenges of linking and accessing secure information systems used by health and care providers.

**Figure 4.**
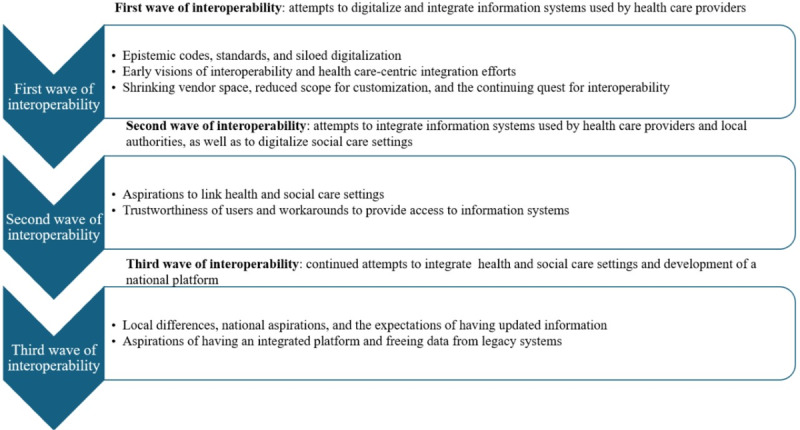
Themes and subthemes characterizing different waves of interoperability.

The third wave, shown in Figure 3, built on the second wave with further iterations of Digital Health and Social Care Strategies (2021 and 2025), rearticulating and expanding the vision of interoperability in response to the limitations encountered in earlier waves. These imaginaries envisioned digitalizing social care providers operating care homes and interlinking their information systems with those used by health care providers. However, national strategies to repurpose the KIS to new missions during the COVID-19 pandemic affected the quality of information in the KISs, creating tensions between local information needs and national aspirations. The ongoing third wave also aims to free data from siloed health and social care systems by introducing a fundamentally new approach centered on developing an integrated platform, reflecting a renewed imagination that seeks to overcome the fragmentation embedded in the existing installed base and reconfigure it to provide more integrated, person-centered models of care. The following paragraphs will examine each wave and its emerging subthemes in detail, tracing how the aforesaid policies as broader imaginaries unfolded as illustrated in Figure 3, their various material and performative effects, and their influence on the development, implementation, and adoption of the KIS. Figure 4 summarizes the waves and the different subthemes within them.

### First Wave of Interoperability

#### Overview

The first wave of interoperability emerged from the siloed digital transformation of GP practices and hospitals in the 1990s, followed by attempts to link these systems during the next decade, reflecting early imaginaries of interoperability that envisioned the electronic sharing of patient information across fragmented health care providers.

At a national level, policy and technology developers attempted to develop an XML-based architecture linking different primary and secondary care providers, representing what they considered technically achievable at the time in advancing these visions [43], primarily enabling technical interoperability through structured electronic data exchange between systems. This was followed by setting up metadata standards and guidance to support the consistent sharing and storage of clinical information at a regional level. Following the success of XML-based interoperability efforts, the Scottish Government launched the SCI (Scottish care information) program in 2000 to provide clinicians with a regional repository for accessing health data across multiple sources, while the Community Health Index ensured consistent patient identification across health care settings.

These initiatives gave rise to a range of sociotechnical and socio-organizational effects at regional and local levels. These included the formation of data-sharing partnerships, the establishment of eHealth strategy and information-sharing boards, and the gradual development of systems such as the KIS, aligned with existing GP coding practices.

The following sections will illustrate how these national and regional efforts, shaped by the constraints posed by the installed base, influenced the trajectory of integration through digitalization in the first wave.

#### Epistemic Codes, Standards, and Siloed Digitalization

GPs in Scotland were early adopters of electronic patient records and, as the primary point of contact for a person’s health care needs, they emerged as gatekeepers of patient data, positioning GP-held data as the foundation upon which early imaginaries around interoperability were built. Subsequently, the data held and shared by GPs became the authoritative narrative of a person’s health history.

*GP surgeries were amongst the first to computerize the records, so they also became gatekeepers of the data and patient records. The expectation was that a GP practice has a long-term relationship with the patient and may be better placed to put something. Once it becomes digital or it’s etched in ink, it becomes the gospel or the truth, whether it is or not*.

[Clinician, Hospital, Lothian]

Widespread uptake of the General Practice Administration System for Scotland (GPASS) among GPs shaped the trajectory for Scotland’s long-term approach to digitalizing and integrating primary and secondary care information systems, embedding a GP-centric installed base that would both enable and constrain future interoperability efforts. The GPASS, developed by a GP in Scotland, was a bespoke, user-built application that was initially designed to record and repeat prescriptions. GPs in Scotland had free access to GPASS, which incentivized GPs to implement GPASS and digitalize their practice. Moreover, the widespread uptake of GPASS, embedded as the installed base, influenced subsequent development of hospital-based IT systems and efforts to integrate systems used in primary and secondary care.

*A GP [name] gave a software to the Scottish Government and said he'd built a hacker system. He was a GP and a computer scientist; he'd built a very small app that just did repeat prescriptions for GPs, and out of that became a programme called GPASS, which was a pretty good system at one time. That definitely colored thinking in Scotland, that there was that commitment to one single system. GPs were never forced to have that system, but they got that for free, which was a big plus, whereas if they went for one of the commercial versions, they had to find the money for that themselves. So that probably colored a lot of thinking, and later they were going to build a hospital system on top of the GP system*.

[Vendor, Technology Developer, United Kingdom]

Policymakers highlighted that GPs were persuaded and encouraged to form user groups to contribute to the development of GPASS, as GPs were the primary source of digital health data. Consequently, networks of GPs formed communities of practice, such as the Scottish Clinical Information Management in Practice (SCIMP), which, through long-term engagement, influenced fellow GPs to digitalize their practices and develop codes for the GPASS. The GPASS, developed on an open application programming interface, was originally designed to function as an administration system and was later extended to include clinical records. GPASS, as with many other information systems used by GPs, was built on clinical coding standards derived from read codes—a coded thesaurus of clinical terms used across the NHS before the adoption of SNOMED CT [11], supporting early semantic interoperability within primary care but not consistently across settings. As a result of the open interface, wide diffusion, and use of common standards, users (GPs) contributed to the development of GPASS as a clinical coding system.

*With the constraints we had on the technology at the time meant that it had to come from the GPs. So, there was certainly a lot of persuasion to try and encourage GPs to add some codes*.

[Policymaker 1, Scottish Government Digital Health and Care, Scotland]

#### Early Visions of Interoperability and Health Care–Centric Integration Efforts

With an enduring and growing need to share up-to-date information to provide better quality care, policymakers prioritized interoperability as the focus of the following national efforts to standardize information sharing across health care providers, consolidating earlier imaginaries into more coordinated strategies, which serve as the installed base to date. Long-term strategies developed by the Scottish Government established an XML-based data-sharing architecture to connect primary and secondary care. Introduced in 1997, this architecture enabled clinical information to be shared electronically between GPs and hospitals. It also aided policymakers and technology developers in developing and implementing records such as the ECS and KIS, to share information from GP records to other health care providers.

*So that would take you back to 2001, when a lot of stuff was created under the Scottish Care Information [SCI] structures, and we are still here with the same architecture. Scotland was quite ahead in terms of developing Extensible Markup Language [XML] schemas for Emergency Care Summary [ECS], Key Information Summary [KIS], SCI store, and that was all done in the early nineties, to late nineties, and about 2006*.

[Policymaker 2, Scottish Government Digital Health and Care, Scotland]

Data models for ECS and KIS emerged from medical records held by GPs, which differed from how other health care providers recorded medical data. To ensure technical and semantic interoperability, policymakers issued guidelines to health IT suppliers in Scotland for developing software that also aligned with GP coding standards, further embedding these standards across the information infrastructure. Early interoperability efforts were intended to enable structured data exchange between providers using systems such as the KIS. Although XML-based integration initially facilitated effective data sharing, complexities and variance in coding health data resulted in multiple inconsistent versions of the KIS, revealing tensions between locally embedded coding practices and national aspirations for semantic standardization across health care settings. Clinicians, technology developers, and policymakers worked to define shared data items and the associated information governance mechanisms for their exchange across health care providers by developing common coding terminologies and establishing regional information-sharing agreements to facilitate data exchange. But commonly agreed standards between the different entities were not developed, and data models for the KIS emerged from how GPs coded medical information. Furthermore, SCIMP developed standardized guidelines for health IT suppliers using medication data models created and refined by a small group of GPs, and it directed suppliers to align their products with the data structures used in ECS and KIS.

*Probably around 2000, just before Scotland decided to create this XML–based interoperability. It had a lot of concrete models for doing a diagnosis or an allergy, and that’s what ECS and KIS were built on. But what it showed with KIS was that it started to creak because, although XML schemas are designed to be extensible, they get quite hard to control because of the complexity of health data. So one of the things that Scotland got right is XML integration, because it was way better than anything around. It was generally a pleasure to use, but they didn't understand how to govern and extend it, and they lost control. So you ended up with like 10 different versions of KIS, and everybody’s doing something different. That was a big lesson for me about the importance and the difficulty of growing your semantics without losing control*.

[Policymaker 1, Scottish Government Digital Health and Care, Scotland]

#### Shrinking Vendor Space, Reduced Scope for Customization, and the Continuing Quest for Interoperability

**Figure 5.**
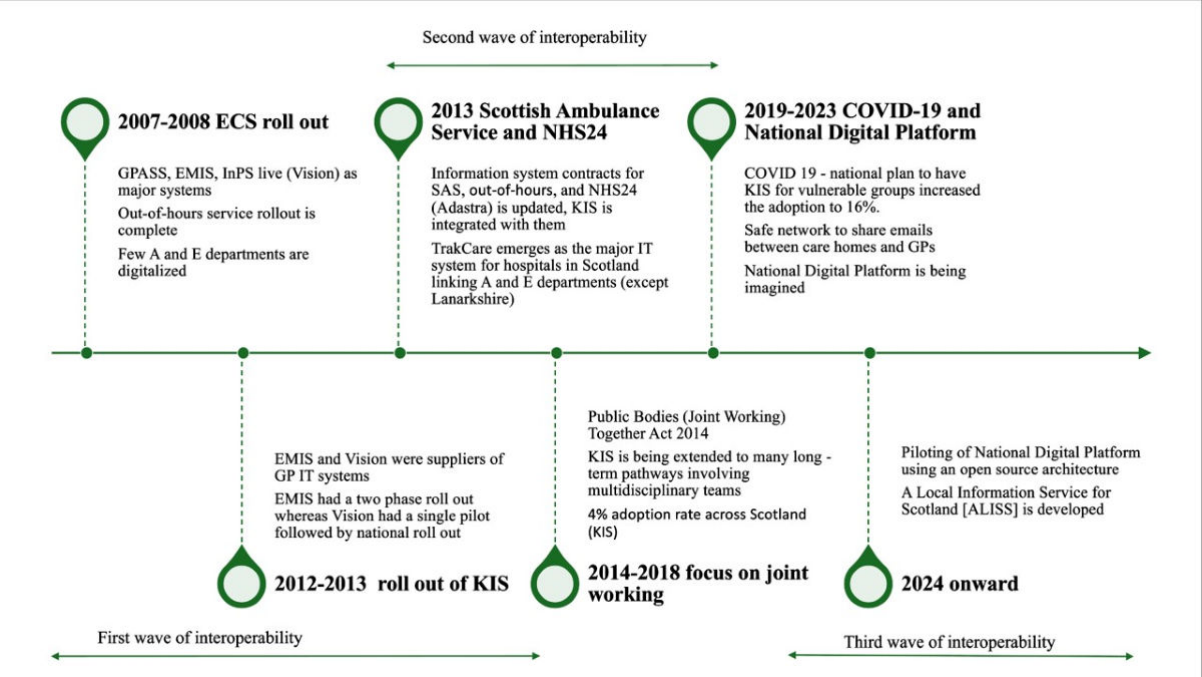
Evolution of KIS. ALISS: A Local Information Service for Scotland; EMIS: Egton medical information system; GP: general practitioner; GPASS: General Practice Administration System for Scotland; KIS: Key Information Summary; SAS: Scottish Ambulance Service.

As the demand for interoperable standards between health care providers grew, policymakers introduced strict compliance requirements for health care IT suppliers, forcing out noncompliant legacy systems and introducing standardized products at a national level, with limited scope for customization to configure to local needs. Figure 5 illustrates how this happened over time regarding the KIS and the three waves of interoperability.

While the KIS was being developed, the GPASS was getting costlier to run and became outdated compared to other off-the-shelf information systems developed for the English market, leading to its replacement by Egton medical information system and Vision systems, through which the rollout of the KIS across GP practices in Scotland was managed. At the same time, out-of-hour services had started to use Adastra, and hospitals were using TrakCare in all health boards, which enabled viewing the ECS and KIS. TrakCare had been in use in Lothian before national-level adoption. Lothian thereby had a customized version, which other boards did not have, and were able to configure the system to their specific needs, compared to the other boards, which were offered a generic and less customizable version.

*A lot of the stuff we want to do in TrakCare, Lothian is in a fortunate position where, unlike other boards, Lothian can do a lot more configuration and development within TrakCare. Whereas the majority of the boards in Scotland are on the Scottish edition, and they're much more reliant on the vendor wanting to do something to customize*.

[Technology lead, NHS Lothian, Lothian]

### Second Wave of Interoperability

#### Overview

The second wave emerged following aspirations to link systems used by health care providers and local authorities after the introduction of the Public Bodies (Joint Working) (Scotland) Act 2014, and to digitalize various social care providers, as illustrated in Figures 3 and 5, reflecting emerging imaginaries of interoperable health and social care systems that extended beyond health care settings.

At the national level, these imaginaries were articulated through policy and standardization efforts. The Health and Social Care Information Sharing Strategic Framework (2014) [44] established the basis for information sharing between local authorities and regional health boards, mobilizing actors around these new expectations of interoperability. Policies and guidelines such as the Digital Health and Social Care Strategy [45] and the Care in the Digital Age report [46] also suggested measures to build digital skills and strengthen the social care workforce, translating these renewed expectations into policy direction.

This wave included nationwide implementation and adoption of the KIS, alongside other national-level efforts to digitalize social care services, as illustrated in Figure 3, reflecting the materialization of earlier interoperability ambitions and a renewed imaginary of more comprehensive coordination across health and care providers. In parallel, a national-level digital platform was being planned and developed by the Scottish Government to integrate data held in different health and care settings using an open-source architecture.

These national-level initiatives gave rise to a range of sociotechnical and socio-organizational effects. As a result of these national-level initiatives, local partnerships and information-sharing boards were established to coordinate data sharing. However, common standards to link health care providers and local authorities had not yet been developed by diverse stakeholders and technology vendors, leading to the extension of the existing health care–centric information infrastructure to include local authorities. These extensions granted read-only access to EHRs for different professionals in different clinical and nonclinical settings and established secure emailing networks to support electronic messaging between health care providers and local authorities.

Formal workarounds, that is, workarounds agreed and promoted by health boards, such as providing proxy access on behalf of the health board, enabled read-only access to EHRs across various nonclinical settings, as pragmatic responses to infrastructural limitations. The different imaginaries and attempts to link information systems used by health care providers with those of local authorities and social care providers, as unfolded in the second wave, are explored further in the subsequent sections.

#### Aspirations to Link Health and Social Care Settings

Attempts to digitalize and link information systems used in health care services in the first wave were followed by attempts to link them with information systems used by local authorities for social work. During the development and implementation of the KIS, there were parallel attempts to develop information-sharing mechanisms and agreements between health care providers and local authorities. Building on earlier policy efforts to improve coordination and enable information sharing, local authorities and health boards were directed to establish local information-sharing partnerships and engage with national-level information-sharing bodies. To support this approach, policymakers created a new Scottish Interoperability Working Group comprising representatives from both sectors to agree on common standards for information sharing between health care providers and local authorities.

However, the interoperability working group struggled to establish common standards due to divergent epistemic coding practices and a lack of collaboration to align them. Health and care organizations relied on distinct information systems tailored to their specific needs. For example, hospitals used data standards and recording practices that varied not only between regions but also from those used by GPs and local authorities. Although stakeholders acknowledged the importance of alignment, they struggled to deliberate and develop collaborative mechanisms to standardize and harmonize these practices. While these policies and guidelines offered potential pathways for coordination, the absence of effective leadership impeded meaningful interprofessional collaboration, constraining the enactment of these imaginaries.

*We spent a lot of time agreeing on data standards and models between councils and hospitals. But we realized that, just a mile away, in the major hospitals, people were being discharged with completely different data standards, and that’s a critical part of it. The thing that we couldn't do was figure out a way to have those conversations with all these different parties to say, “Look, is there a place we can start? This is our data model, this is yours, and how do we grow these?” But it kind of fell apart*.

[Technology developer, Interoperability Working Group, Scotland]

Electronic networks initially designed to connect health care entities were later extended to include local authorities through information-sharing agreements with health boards, to pursue the expectations of digitally interlinking these fragmented health and care providers. However, this partial extension meant that social workers from local authorities were only able to receive the KIS in the form of a PDF and communicate any updates using a template tailored to their role by the health board. These extensions resulted in generating different material forms of KIS, accessible to different clinical and nonclinical entities through their information systems, illustrating how constraints imposed by the installed base shaped the extent and form of interoperability achievable across settings.

However, social care providers, operating care homes, remained excluded, as their digital transformation was still underway and uncoordinated, with policymakers highlighting the resulting diversity in the adoption and use of digital technologies. Documents published by Care Quality, a national body assessing the quality of care, distinguish social care providers based on their ownership, including local authorities, health boards, private, and voluntary services. These different care providers had varying levels of digital maturity, with larger organizations generally possessing more advanced digital tools and IT support. In contrast, smaller organizations had limited digital capabilities and resources. For example, some of the staff employed by the council had email accounts to communicate and share patient information with GP practices using a secure emailing network. But many care homes, run by private and voluntary services, either lacked email addresses or were not recognized as trusted entities within the secure Microsoft email network used by clinical professionals, which limited their ability to access KIS in a digital format.

*When it comes to social care, there are challenges around the range, size, and number of providers. When you're talking about information flow between the NHS, the local authority, and the social care providers, that implies a lot of interoperability, a lot of information governance. So, imagine all those things in a health setting, they're multiplied when it comes to social care and then add into the mix that your IT systems are less well developed in a lot of social care settings. Not every social care setting, because when we're talking about independent third sector providers, they vary in size from the one- and two-person operation right up to hundreds and thousands of members of staff with an IT department, but many won't have that*.

[Social Care, Scottish care, Scotland]

#### Trustworthiness of Users and Workarounds to Provide Access to Information Systems

**Figure 6.**
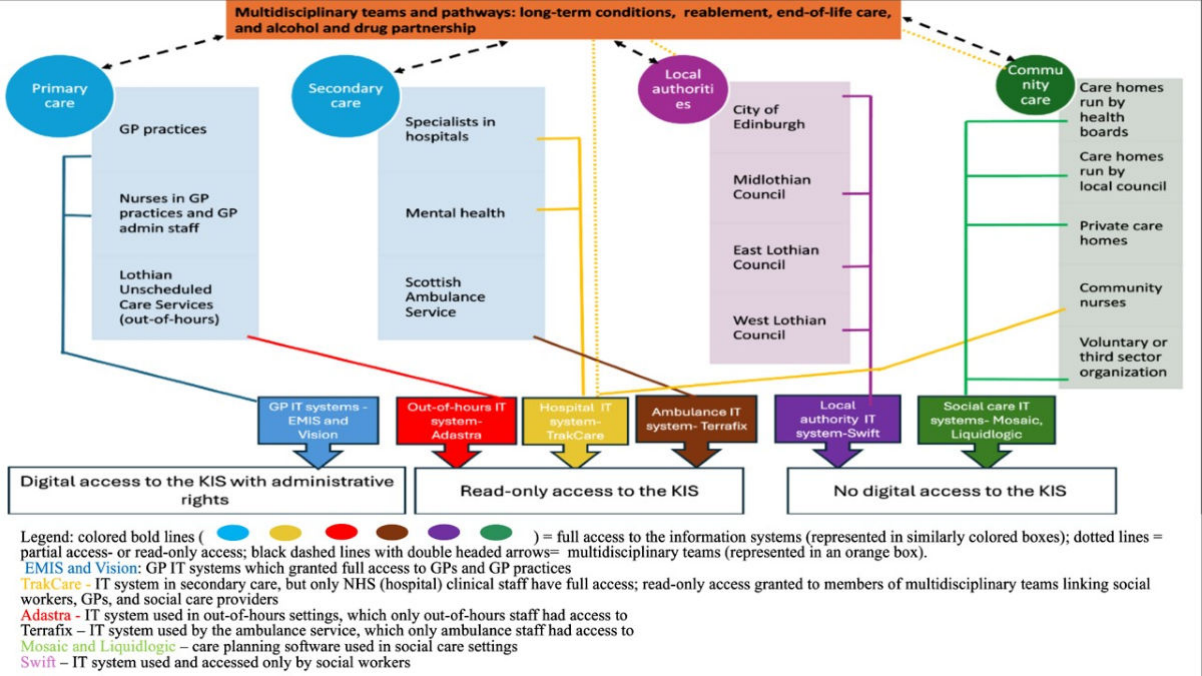
Information systems used by health and care entities in Lothian to access the KIS. GP: general practitioner; KIS: Key Information Summary; NHS: National Health Service.

Figure 6 illustrates how accessing the KIS varied across clinical and nonclinical settings, depending on the information system used, the user’s role, and the care pathways involved. The KIS was developed to share key information from GP settings to out-of-hour services, ambulance services, specialists in hospitals, social workers, and caregivers. Earlier attempts to enable interoperability between information systems used in health care settings enabled electronic sharing of documents and EHRs among health care providers. As a result, the KIS was accessed by out-of-hour services, hospitals, and ambulance services, using their respective information systems. GPs had administrative rights to edit and share the KIS using GP IT systems, as the KIS contained a summary of data held in GP records, whereas clinicians in hospital settings, out-of-hour settings, and ambulance services had read-only access. Similarly, GPs and clinicians in hospitals had restricted access to systems used by out-of-hour services. Social care providers used information systems that were not connected to health care systems or to the council’s system. This was because the systems were designed for specific professional use and operated under separate information governance frameworks and access permissions that limited legal and organizational interoperability. However, as imaginaries of integrating health and social care entities evolved and due to constraints imposed by the installed base, regional health boards provided social workers from local councils and a small number of social care providers involved in MDTs with read-only access to TrakCare to view the KIS. Initially, nonhospital staff did not have access to TrakCare due to strict access controls, but formal workarounds agreed by health boards enabled the extension of the KIS to social workers and social caregivers by providing proxy access through TrakCare.

Formal workarounds were used by regional health boards despite having strict administrative restrictions on access to different clinical systems to extend the health care–centric information infrastructure to nonclinical settings and provide nonclinical members access to the KIS. These arrangements constituted partial and ad hoc adjustments to address challenges around organizational and legal layers of interoperability, rather than a transformation of the underlying infrastructure, while still conferring legitimacy for such access. Proxy access to TrakCare enabled temporary access on behalf of the health board, allowing a small number of care home managers and senior social workers involved in long-term care pathways to access the KIS, overcoming existing legal and organizational restrictions around access, but it created uneven access and increased administrative workload. For instance, social workers in the Dementia Post Diagnostic Service—a care pathway that supports newly diagnosed dementia patients in developing their future care plans—were granted access to TrakCare to ensure that information about future care in the KIS remained relevant and to communicate updates to the GP. Regional health and care partnerships, such as the EHSCP, created templates for social workers involved in such MDTs and care pathways to share relevant information regarding a person’s future care with GPs. Not everyone in the team had access to TrakCare, meaning that senior-level social workers with proxy access had to share updates on behalf of other members of the team, which increased their workload.

*But not everybody can look at TrakCare because not everybody has TrakCare accounts. There are a lot of my colleagues who do not have track accounts, people who are coordinators*.

[Social work, Lothian]

### Third Wave of Interoperability

#### Overview

The third wave, as with the second wave, aimed to link health and social care services with evolved expectations around interoperability for integrated care and tackling persisting challenges around information sharing between health and social care entities. At a regional level, health boards such as Lothian further extended the secure email network developed during the second wave to include all social care entities, as many care providers had access to digital tools for electronic communication and care planning, as illustrated in Figure 5.

National policies around integrated care also evolved to include a person-centric approach, ensuring that people’s preferences and choices guide care planning and clinical decision-making. As a result, the development of the national digital platform initiated by the Scottish Government progressed. This national platform, being developed to integrate multiple EHRs and data from different health and care settings using an open-source architecture, represented a more radical reimagining of interoperability beyond incremental extensions of the installed base. Additionally, an open-access search engine for all care services (ALISS [A Local Information Service for Scotland]) was developed by alliances of social care providers, as illustrated in Figures 3 and 5.

During COVID-19, top-down efforts, that is, directives from government authorities, accelerated the adoption of the KIS. These national directives mandated the use of the KIS to identify vulnerable groups and introduced changes to its governance. Specifically, the special notes field within individual KIS records indicated that the KIS had been created as part of the COVID-19 protocol, and the consent model shifted from an opt-in approach to implied consent, allowing GPs to consent on behalf of individuals for the sharing of their KIS. At the individual level, this led GPs to generate KIS records for patients who previously did not have one, with limited substantive care-planning content beyond the COVID-19 marker used to identify vulnerable patient groups.

These top-down strategies during the pandemic played a significant role in shaping the expectations of having a national-level platform and sharing real-time information. This will be explored further in the subsequent sections.

#### Local Differences, National Aspirations, and the Expectations of Having Updated Information

Top-down government approaches during COVID-19 directed the KIS to be used to identify vulnerable groups, diverting from its original intended use and breaching existing consent mechanisms, resulting in an increase in recorded KIS entries across Scotland from 4% to 30%, as illustrated in Figure 5. The KIS was designed to have an opt-in consent model, allowing GPs to identify people who would benefit from having a KIS to support their care needs, ensuring explicit permission was obtained before sharing. However, these top-down approaches disrupted the information quality in the KISs as the policies were not aligned with the realities of how the KIS was used by different clinical and nonclinical entities to share Future Care Plans, revealing tensions between national imaginaries and situated use. Following the national mandate, users were unable to establish a systematic method for retrieving KISs created before COVID-19 that documented individuals’ care preferences. Users with access to TrakCare in hospital settings were able to look at other sources of information, such as the ECS, to identify people who would benefit from an updated KIS and communicate their expectations with the GPs. While such approaches helped identify those who would benefit from a few practices, there was no national-level strategy to monitor and target the quality of information in the KIS post-COVID-19. The following quote illustrates the challenges and unanticipated consequences of top-down strategies shaping the use of KIS.

*Post-COVID, because consent was implied for everybody, we ended up with thousands of people for whom consent had been implied. But there was no data there, secondary care could at least see things like the past medical history and anything that was coded but it made it very difficult to then understand who still needs to get a KIS, who’s got one, who’s got a review date coming up, who’s got a KIS but doesn't have resuscitation status recorded, or who has resuscitation status but no KIS. But then post-COVID, that data became meaningless because everyone had a KIS, and it didn't differentiate between people who had a meaningful KIS with an anticipatory care plan with good special notes, and those who had just the text stating “Created as part of COVID-19”, and that made the monitoring and targeting of the KIS really difficult. I don't think we've still got a solution to that, to be honest*.

[Clinician 3, GP Practice, Lothian]

The lack of a national strategy for retrieving KIS records containing Future Care Plans resulted in numerous localized initiatives, which included using social workers from local authorities and care home managers to provide relevant information to update the KIS, illustrating bottom-up adoption strategies to address policy gaps. Despite initial hesitance from clinicians to extend access to clinical information systems to nonclinical settings, communities of practice such as the EHSCP (regional health and social care partnership) continued engaging with social workers and care homes to assist in providing GPs with relevant information to update the KIS. EHSCP provided training to care home staff and social workers to support GPs in updating the KIS and mobilizing new actors into the infrastructure. Initially, a few clinicians were hesitant to involve social workers and care workers in contributing to or accessing the KIS, as GPs were the gatekeepers of the data held in the KIS, due to differences in coding practices across clinical and nonclinical settings. However, the secure emailing network developed during the second wave was extended to all social care providers to share updates regarding a patient with the GP practices, expanding participation within the existing infrastructure. EHSCP evidenced the benefit of extending access to the KIS across different care settings, showing an increased number of updated resuscitation records and reduced unplanned hospital admissions, thereby reducing the hesitancy among clinicians to involve social workers and social care workers, reinforcing the value of extending access. This process also involved building the confidence of social workers and care workers in using the KIS for Future Care Planning by cultivating a sense of ownership through frequent training sessions.

*We had a lot of hesitation from clinicians around that they did not see that support workers would have a role in informing the information that goes into a KIS, or in accessing it. There was a lot of hesitance at first, but as we started to show the benefits, that waned and it became more accepted and that was really interesting, but it also meant that we had to build up the confidence of social care staff to be able to participate in that way and to be able to use the KIS and, have as much ownership over it*.

[Social work, EHSCP, Lothian]

#### Aspirations of Having an Integrated Platform and Freeing Data From Legacy Systems

Decades of efforts to enable interoperability had resulted in a complex network of information systems using specialized channels for point-to-point communication, relying on role-based access control arrangements to access information systems designed for health care systems. This limited data sharing with nonclinical entities for providing patient-centric care. Following its initial implementation, the KIS had been extended to include various care pathways as the expectations of providing integrated care and interprofessional working evolved, iteratively expanding the infrastructure to include new members. While these extensions aimed to better share information with different service providers to provide patient-centric care, the fragmentation of legacy systems and existing information governance mechanisms hindered data sharing. The following quote illustrates the evolving expectations of service providers and users regarding data sharing across health and care settings, and the inadequacy of current information-sharing and consent mechanisms to meet them, underscoring the need for systemic reform.

*We must see how we got here in the last 15 years or so. Everything was around primary care, and then from 2012-14 we started working together. The KIS is almost 15 years old. If you see from where it evolved, the landscape has changed drastically, and the KIS is still stuck in it. Back then, people were also hesitant to share their information. But now things have changed, and people interact with others. If you take the cancer care pathway, then the third sector would be involved. They know this person, and when we ask if we could share your information with them, then the person asks, “Wasn’t that done already?” It is sad that our way of working has changed, people’s ideas about data sharing have changed, but the software and its idea of consent don’t help it. These are obstacles around information governance we need to answer. It’s time for an overhaul*.

[Policymaker 1, Scottish Government Digital Health and Care, Scotland]

In contrast with existing information sharing practices, where GPs are custodians of patient data, a forward-looking group of policymakers and technology developers, reflecting on the limitations of the existing system, envisioned a national-level platform to support patient-centric care and interoperability by positioning citizens as gatekeepers of their health data. This contrasting approach emerged from limitations of the existing information governance mechanisms and information sharing practices, which did not evolve in tandem with the expectations of providing integrated care linking various health care, social work, and social care providers. Consequently, the development of a nation-level platform using an open-source architecture was commissioned in 2018 by the government and piloted in 2022. However, neither has there been a strategy or plan for national rollout, nor has there been an understanding of how the KIS will be integrated with other EHRs used in hospitals for Future Care Planning. Such a platform would require existing software vendors to provide data in a shareable format to the core data repository held nationwide. Restructuring the KIS would require understanding the current information needs of different users and the means to gather them from disparate information systems, which the existing information-sharing mechanisms restrict. Moreover, different health boards and care providers maintain distinct contracts with various technology suppliers, resulting in a fragmented and asynchronous procurement system. Harmonizing these contracts would require renegotiating existing agreements and synchronizing their terms, supported by strong leadership to coordinate and mobilize necessary resources and actors.

*It'd be very hard to integrate the NDP with ECS or KIS. It’s pretty hard to change ECS and KIS for anything. So, the idea that you have access to all this additional information and finding a way of putting that into ECS and KIS in a logical way is hard. Where it could work really well would be if you have a tool that’s on the NDP, that, as one of its spokes, could have colleagues write and read information, and then it’s essentially available. But yeah, I think as ECS and KIS are currently constructed, we'll have a hard enough time*.

[Clinician and policy lead for Scottish Government, GP Partner, Lothian]

*Being able to harmonise all those singular contracts is an extremely difficult thing to do. It would be extremely resource-intensive. It would require all the boards to agree on what that harmonised contract looks like, and that’s honestly a challenge. And one of the biggest challenges is that no one person or organisation is well-suited to be the person who says, “Let’s bring this together and let’s deliver this together*.”

[Policymaker 3, Scottish Government Digital Health and Care, Scotland]

## Discussion

### Summary of Findings

Drawing upon the sociology of expectations and the concept of information infrastructures, this study demonstrates how the quest for interoperability has been iterative, resurfacing with evolving expectations of providing integrated care through a series of waves. Each wave was characterized by attempts to link disparate information systems used by different health and care providers, yet each wave was also constrained by existing information-sharing practices and the installed base, which limited what could be achieved in practice and resulted in only partial forms of integration. These limitations, in turn, contributed to the rearticulation and intensification of interoperability expectations in subsequent waves. The first wave included the interlinking of siloed health care services to aid data sharing between the various primary and secondary care providers. The second wave represented the interlinking of health care providers with local authorities and attempts to digitalize social care services to provide integrated care. The ongoing third wave is characterized by a range of concurrent regional and national efforts to connect health care and social care services to support person-centric models of integrated care, reflecting emerging and radical new imaginaries that seek to shape future integration and interoperability efforts.

Each wave mobilized the creation of material artifacts emerging in the form of new digital technologies or the extension of existing digital technologies to new settings to interlink health and care. These digital technologies shaped organizational practices, embedding new routines and gradually forming a revised installed base into which subsequent technological changes had to be integrated. National-level mechanisms to govern and standardize information sharing facilitated the development and adoption of networks and architectures that interlink information systems across health and care providers, along with the development and adoption of EHRs, such as the KIS, to share information across different entities. Adoption of the KIS varied across health and care settings based on users’ information needs, coding practices, and configurability to local settings. Workarounds at regional and organizational levels facilitated data access and sharing across different clinical and nonclinical settings, with communities of practice supporting digital transformation and embedding digital technologies in local settings as expectations of interoperability and integrated care evolved. These efforts were crucial in extending the health care–centric information infrastructure to social care settings, enabling information sharing to support integrated care using digital technologies.

Different tensions emerged as waves progressed to integrate different health and care providers. In the first wave, these contentions centered on technical and semantic interoperability, particularly codeveloping and agreeing upon shared coding standards among health care providers such as GPs and hospitals; in the second wave, tensions increasingly concerned organizational and legal interoperability, including information governance arrangements, between health care entities and local councils; and in the third wave, between health and social care providers. While the third wave was also characterized by efforts to free data from siloed health and care information systems, there were tensions between having a national-level platform and the deeply embedded nature of the installed base, comprising legacy systems and established practices, alongside the challenge of managing distinct and asynchronous vendor contracts across organizations.

### Strengths and Limitations

This study, using the BoAP and TPOM frameworks, helped retrospectively explore the development, implementation, adoption, and use of the KIS in different settings. Empirical case studies exploring the implementation, adoption, and use of digital health technologies such as EHRs have largely focused on short-term episodic studies [47,48]. In this regard, this research represents a novel attempt to study regional information infrastructures integrating health and social care, using a longitudinal inquiry across multiple settings in which a digital technology was conceived, developed, implemented, and adopted as part of the infrastructure. Existing research on the long-term evolution of information infrastructures often encounters challenges in balancing analytical depth and breadth. These challenges are further compounded by the scale and complexity of such infrastructural projects, which makes it inherently difficult to present a sufficiently rich yet analytically concise account of their development, implementation, and use across multiple contexts. This study addresses these challenges by offering a broad, longitudinal analysis of infrastructure development using the KIS as a case study, tracing its development, implementation, and adoption over time to understand infrastructural growth. However, in doing so, it also inevitably encounters similar tensions between depth and breadth. This study is also limited by its reliance on retrospective accounts, which are vulnerable to recall bias and lack real-time observation of technology development, implementation, and adoption, reducing contextual depth and credibility. While this study concludes with an analysis of developments to date and does not extend to future infrastructural changes, it nevertheless offers theoretical insights that advance our understanding of infrastructural growth.

This research aimed to explore the trajectory of health and social care integration from the perspective of participants from Lothian, which may limit transferability to other regions due to variations in how integrated care using digital technologies is delivered across Scotland. As this study was conducted in Scotland, where health care is publicly funded and centrally governed with regional autonomy, the findings may have limited transferability to contexts with different governance arrangements and health systems. They reflect Scotland’s distinct trajectory of technology adoption and policy development, which may not be directly comparable elsewhere. However, the theoretical insights developed in this study can inform broader analyses of how orchestration efforts at micro, meso, and macro levels enable interorganizational information sharing for integrated care, and how information infrastructures evolve through successive concerted efforts in the form of waves to connect diverse health and care providers. The development of information infrastructures integrating health and social care is a long-term, distributed, and inherently uneven process involving multiple actors working on different components over extended periods. The Scottish case is particularly useful in this regard, as it illustrates the broader difficulty of achieving fully interoperable information infrastructures integrating health and care providers, even in contexts with sustained national-level investment and coordination. It also shows how such infrastructures evolve in response to shifting expectations of interoperability and integrating health and care providers, and the practical constraints encountered in their realization, highlighting integration as an ongoing and structurally contingent process rather than a finite accomplishment. These insights suggest that efforts to integrate health and social care through digitalization are unlikely to succeed through top-down, one-off solutions alone, but instead require sustained alignment between national strategies and locally embedded practices, heterogeneous organizational contexts, and settings having varying levels of digital maturity.

### Integration of Findings With the Literature

This study supports existing research that conceptualizes expectations around technological innovations as temporal phenomena, while also contributing to scholarship on the temporal patterns associated with infrastructure development [20,21,49]. Findings illustrate how the pursuit of interoperability within the long-term generic vision of integrated service provisioning is iterative, emerging, and reemerging in response to evolving care needs but continually constrained by existing information-sharing practices and information infrastructure across successive waves. The metaphor of waves used in this study captures this temporal resurgence of renewed imaginaries around interoperability, reflecting how scholars have traced technological paradigms and trajectories to examine the interactions between expectations, incremental innovation, adoption, and the long-term evolution of incomplete utopian projects [16,49-51]. These findings are consistent with the literature highlighting the temporality of expectations following successive iterations of promise and disappointment [20,21,49].

This study further builds on the sociology of expectations literature, which emphasizes that expectations have both material and performative effects. The findings demonstrate how evolving imaginaries of interoperability and integrated care unfolded over time in distinct waves, with each wave seeking to address the constraints imposed by the de facto information-sharing arrangements to provide integrated care, resulting in a series of material and performative effects. Each wave led to the introduction or extension of standardized technologies into new settings, materializing evolving expectations to integrate health and care providers. However, these extensions required distinct mechanisms for local adoption. As a result, roles, information needs, governance structures, and modes of interprofessional collaboration were reconfigured to translate these expectations into practice. For example, nonclinical professionals (eg, social workers and care workers) were required to engage with clinical information systems to exchange information with health care providers within a health care–centric infrastructure, illustrating how evolving expectations produced both material changes in technologies and performative changes in everyday practices.

These findings also align with the literature in asserting that such long-term utopian projects evolve over various short-term iterative developments, achieving partial realizations of a generic vision [5,16]. These short-term developments and achievements depend on the installed base and its flexibility or hostility in supporting incremental evolution [3,5,6,21]. For instance, the evolution of information infrastructures connecting health and social care providers unfolded over more than two decades, with each successive wave extending the range of actors involved and the activities supported as imaginaries of interoperability and integrated care evolved. Throughout this process, the health care–centric information infrastructure was incrementally extended across different health and care settings, necessitating workarounds at regional and organizational levels to enable its effective adoption and integration within local settings. These findings resonate with existing scholarship, which posits that long-term infrastructural transformations are realized through a series of incremental developments shaped by the constraints of the existing installed base [3,6,16].

By studying the efforts of multiple dispersed communities to effect changes in the growth of information infrastructures integrating health and care providers, this study extends and contributes to the literature on orchestration and the growth of information infrastructures [3-6]. It demonstrates that certain expectations for change require more substantial transformations (eg, developing and implementing a national-level network linking diverse service providers), necessitating the coordinated involvement of a wider range of actors, whereas certain changes can be realized through local enactments (eg, workarounds and contextual adaptations), consequently unfolding over extended temporal horizons. Tracing this evolution, this study further shows that information infrastructures integrating health and social care providers evolved through incremental extensions, enabling the inclusion of new members from diverse settings as expectations of integration evolved. In the realm of infrastructural studies, Star and Ruhleder [4] introduce the concept of membership, which emphasizes learning and developing implicit familiarity with infrastructural artifacts and organizational arrangements to be a part of the infrastructural ecosystem. This research shows that membership and its incremental extension to new groups, shaped by organizational affiliation, professional role, and the trust established within specific communities of practice, created the affordances that enabled users to meet their information needs using digital technologies. Different professionals engaged with specific digital tools and systems available to them based on their organizational affiliation and role as the infrastructure grew to meet evolving expectations of integrated care. Existing members trusted clinical entities such as GPs from out-of-hour settings and specialists from hospitals due to their shared professional expertise and the early integration efforts that established common coding practices and standards that shaped the information infrastructure. Nonclinical actors (eg, social workers and care workers) gained trust through participation in MDTs, where collaboration toward shared goals legitimized their involvement. Membership did not mean complete access or rights, but created different access points to engage with the infrastructure to meet their information needs.

Membership determined by organizational affiliations, coupled with sociotechnical and socio-organizational capabilities to access and share information, created affordances. Affordances, in the field of information systems, are possibilities for action that emerge from the relationship between technology and the goals of users [52]. Recent theoretical contributions in the field have highlighted the relation between sociomateriality, environment, professional boundaries, and organizational limits in shaping agency to initiate practice or action [52-54]. However, the remit of these studies is limited to a single organization or to health care settings (Ibid). This work highlights the different formal workarounds used to accommodate health and social care professionals with different institutional affiliations and levels of digital maturity to access the information infrastructure. Though the scope of access and use varied by role, the incremental extension of the infrastructure to different settings enabled members from nonclinical as well as clinical settings to participate in standardized information sharing using digital technologies. These instances illustrate how information infrastructures evolve over extended periods, with certain expansions facilitated through local enactments by using formal workarounds. This process incorporated new members, creating affordances that enabled them to initiate practices or actions within their organizational and professional contexts to carry out their specific responsibilities.

Scholars examining the role of communities of practice in digital transformation highlight how these communities facilitate knowledge sharing and help enhance members’ digital skills and literacy [54,55,56]. However, existing studies largely focus on the work of single, homogeneous communities of practice composed of heterogeneous members (Ibid). By exploring the long-term trajectory of integrating health and social care providers through digitalization, this study adds to the literature by showing how heterogeneous communities of policy makers, implementers, developers, and users shaped the growth of information infrastructures through successive concerted efforts. Different communities of practice, across various times and spaces, assisted and enabled fellow members from multiple organizations to share knowledge, address persistent problems, and codevelop mitigating strategies, as expectations of integration evolved. Users in different settings developed the skills and resources to use these new digital technologies and participate in the digital network. In all the waves, communities of practice emerged to assist and support users, to bridge the gap between national aspirations, local and regional ways of interprofessional working, and build sociotechnical capabilities. Examples include SCIMP influencing the development and adoption of GPASSs in the first wave, EHSCP facilitating the adoption of the KIS by engaging and training social care workers and social workers in the second and third waves. These examples illustrate how heterogeneous communities of practice, through their sustained engagement in knowledge sharing, not only enabled the adoption of standardized digital technologies in local settings across different times and contexts but also extended information sharing beyond formally permitted boundaries, driven by their recognition of the practical needs of different actors. In doing so, they contributed to the development and evolution of the information infrastructure.

### Implications for Policy and Practice

This research is timely to inform policy and practice around the sociotechnical and socio-organizational installed base that develops as expectations around interoperability evolve through multiple national and regional efforts. It illustrates that long-term infrastructural change emerges through a series of short-term developments, each necessitating coordinated mobilization of actors and resources. This study underscores the importance of balancing top-down policy initiatives with a pragmatic understanding of local capabilities and practices. Top-down initiatives that are misaligned with local practices may become disruptive over time, as exemplified by the repurposing of the KIS to identify vulnerable patients during the COVID-19 pandemic. National policies and procurement strategies must therefore align with the realities of system use and the affordances of users at a regional and local level.

As national initiatives pursue achieving interoperability and integrating health and care providers, it is crucial to examine how organizations and actors respond to and implement these changes locally. Organizations and users adopt different approaches to integration depending on their context—organizational structures and needs, user skills, information-sharing practices, work routines, and existing information systems. Achieving interoperability requires a long-term, system-wide perspective, prioritizing stakeholder engagement and collaboration across various stages of technology development, implementation, and adoption to embed digital technologies in diverse health and care settings. Interoperability strategies and policies to support integrated care and facilitate interorganizational information sharing must recognize the diversity of system use, workflows, and priorities of users. Different stakeholder groups hold varying expectations regarding the integration of health and social care through digitalization, possess differing levels of digital maturity, and consequently require varying levels of support. Encouraging and engaging local communities of practice can facilitate integration and interoperability by identifying these challenges, providing cross-organizational support, sharing knowledge, and promoting the adoption of digital technologies within local settings.

### Conclusions

The integration of health and social care services using digital technologies is an ongoing, long-term process that grows through successive waves of change, necessitating sustained planning and recognizing the complex installed base that evolves. Information infrastructures integrating health and social care providers in Scotland did not follow a linear, “big-bang” model of implementation. Instead, it emerged gradually through the mobilization of responses to evolving expectations of interoperability and integrated care. These expectations exposed the limitations of existing fragmented modes of information exchange, leading to the development of more standardized technologies. However, the realization of these imaginaries around integration through digitalization was constrained by existing workflows, coding practices, and legacy systems. As a result, integration efforts unfolded iteratively in which new technological solutions were continually shaped by, and adjusted to, the constraints of the installed base. Users and communities of practice played a central mediating role in this process, enabling adoption across different contexts and demonstrating that interoperability is fundamentally a sociotechnical and socio-organizational achievement rather than a purely technical one. This study theorizes how orchestration efforts at micro, meso, and macro levels mobilized different stakeholders and resources to integrate health and care services using digital technologies through successive concerted efforts. These theoretical insights can guide future research by helping to explain long-term infrastructural growth and the complex interplay of macroenvironmental, sociotechnical, and socio-organizational factors that shape the evolution of information infrastructures to integrate health and social care. From a policy perspective, efforts to integrate health and care through digitalization should take a long-term view, recognizing the diverse information needs and capabilities of users. Sustained support is essential to enable adoption, promote collaboration, and engage communities of practice in facilitating knowledge sharing and cross-organizational coordination. These insights can inform future research and policy by elucidating strategies to facilitate the progressive development of information infrastructures for integrated care delivery.

## Abbreviations

ALISS: A Local Information Service for Scotland
BoAP: Biography of Artifacts and Practices
ECS: emergency care summary
EHR: electronic health record
EHSCP: Edinburgh Health and Social Care Partnership
GP: general practitioner
GPASS: General Practice Administration System for Scotland
HL7: Health Level Seven
IJB: Integrated Joint Board
KIS: Key Information Summary
MDT: multidisciplinary team
NHS: National Health Service
SCI: Scottish care information
SCIMP: Scottish Clinical Information Management in Practice
STS: science technology study
TPOM: Technology, People, Organization, and Macroenvironment
